# Incidence of low central venous oxygen saturation during unplanned admissions in a multidisciplinary intensive care unit: an observational study

**DOI:** 10.1186/cc5144

**Published:** 2007-01-09

**Authors:** Hendrik Bracht, Matthias Hänggi, Barbara Jeker, Ninja Wegmüller, Francesca Porta, David Tüller, Jukka Takala, Stephan M Jakob

**Affiliations:** 1 Department of Intensive Care Medicine, University Hospital Bern, University of Bern, Freiburgstrasse, CH-3010 Bern, Switzerland

## Abstract

**Introduction:**

It has been shown that early central venous oxygen saturation (ScvO_2_)-guided optimization of hemodynamics can improve outcome in septic patients. The early ScvO_2 _profile of other patient groups is unknown. The aim of this study was to characterize unplanned admissions in a multidisciplinary intensive care unit (ICU) with respect to ScvO_2 _and outcome.

**Methods:**

Ninety-eight consecutive unplanned admissions to a multidisciplinary ICU (median age 63 [range 19 to 83] years, median Simplified Acute Physiology Score [SAPS II] 43 [range 11 to 92]) with a clinical indication for a central venous catheter were included in the study. ScvO_2 _was assessed at ICU arrival and six hours later but was not used to guide treatment. Length of stay in ICU (LOS_ICU_) and in hospital (LOS_hospital_) and 28-day mortality were recorded.

**Results:**

ScvO_2 _was 70% ± 12% (mean ± standard deviation) at admission and 71% ± 10% six hours later (*p *= 0.484). Overall 28-day mortality was 18%, LOS_ICU _was 3 (1 to 28) days, and LOS_hospital _was 19 (1 to 28) days. Patients with an ScvO_2 _of less than 60% at admission had higher mortality than patients with an ScvO_2 _of more than 60% (29% versus 17%, *p *< 0.05). Changes in ScvO_2 _during the first six hours were not predictive of LOS_ICU_, LOS_hospital_, or mortality.

**Conclusion:**

Low ScvO_2 _in unplanned admissions and high SAPS II are associated with increased mortality. Standard ICU treatment increased ScvO_2 _in patients with a low admission ScvO_2_, but the increase was not associated with LOS_ICU _or LOS_hospital_.

## Introduction

Tissue hypoperfusion contributes to the development of organ dysfunction [1]. Consequently, tissue perfusion should be monitored in patients at risk. Unfortunately, routinely monitored variables, such as blood pressure, heart rate, urine output, blood gases, or cardiac filling pressure, do not necessarily reflect the adequacy of tissue perfusion [2]. Mixed venous oxygen saturation (SvO_2_) and central venous oxygen saturation (ScvO_2_) have been proposed as better indicators of adequacy of oxygen supply. SvO_2 _can predict outcome in cardiovascular surgery [3], severe cardiopulmonary disease [4], and septic shock [5]. Controversies exist about whether ScvO_2 _can be used as a surrogate for SvO_2 _[6,7]. Venous O_2 _saturation values differ among organ systems due to variable regional oxygen extraction. It is therefore reasonable to conclude that the absolute value of venous oxygen saturation depends on the site of measurement [8]. Several conditions such as redistribution of blood flow (for example, in shock, severe head injury, general anesthesia, and microcirculatory disorders) may affect the relationship between ScvO_2 _and SvO_2 _[9,10]. Nevertheless, although ScvO_2 _reflects mainly the relationship between oxygen supply and demand from head, neck, and upper extremities only [8], it correlates reasonably well with concomitantly measured SvO_2 _values [6,11]. In high risk surgical patients, low ScvO_2 _values are associated with increased rates of perioperative complications, but not with mortality or length of hospital stay [12]. However, in the study of Pearse and coworkers [12], observational data from a randomized, controlled interventional trial were used. Although a carefully defined treatment protocol was applied in their study and goals for arterial oxygen saturation, hemoglobin, heart rate, mean arterial pressure, serum lactate, and urine output were the same in all patients, intravenous fluid administration was guided by central venous pressure in one group of patients, whereas in others fluid management was guided by stroke volume and supplemented with low-dose dopexamine. With such a design, the predictive nature of ScvO_2 _may relate both to the initial cardiovascular dysfunction and to subsequent attempts to correct it.

In patients with severe sepsis or septic shock admitted to the emergency department, ScvO_2_-guided hemodynamic optimization has been shown to reduce mortality [13]. Whether other patient groups may also profit has not yet been determined. Because central venous catheterization is frequently performed in unplanned intensive care unit (ICU) admissions, routine screening for low ScvO_2 _could easily be performed, and ScvO_2_-guided optimization, if proven beneficial, could be established early during the ICU stay. The goal of this study was to evaluate whether ScvO_2 _at admission and six6 hours later is associated with outcome in patients requiring unplanned admission to the ICU.

## Materials and methods

This study was approved by the Ethics Committee of the Canton of Bern, Switzerland, and deferred written informed consent was obtained from patients where possible or from a close relative. All unplanned admissions between October and December 2004 were screened for inclusion and exclusion criteria in a multidisciplinary 30-bed ICU.

The inclusion criterion was either the presence of or a clinical indication for a central venous catheter. Exclusion criteria were a contraindication for a central venous catheter and refusal of blood products. The clinical indication for a central venous catheter was determined by the attending physician, and patients were enrolled in the study only if the first blood sample from the central venous catheter was obtained within the first two hours after ICU admission.

### Protocol

All patients were treated according to standard practice for the ICU. Protocols for hemodynamic treatment, weaning from mechanical ventilation, sedation and analgesia, anticoagulation, and management of blood glucose and potassium were used in all patients where applicable. Whenever possible, a central venous blood sample was obtained immediately after ICU admission, or two hours afterward at the latest, for the determination of oxygen saturation, blood gases, and hemoglobin. Blood gas analyses were performed by intermittent blood sampling and co-oximetry (ABL 725; Radiometer A/S, Brønshøj, Denmark). For all blood gas analyses, the same automated blood gas analyzer was used. PO_2 _(oxygen pressure) was corrected for actual body temperature, and inspired fractional oxygen concentration (FiO_2_) was recorded concomitantly. If an arterial catheter was in place, an arterial blood sample was taken at the same time for the same analysis. Sampling from both sites was repeated six hours later. Persons involved in the treatment of the patients were blinded to the results obtained from the central venous blood.

### Data collection

For data analysis, the patients were divided into five groups according to the main clinical problem that necessitated admission to the ICU: sepsis (*n *= 26), cardiocirculatory dysfunction/failure (*n *= 12), respiratory dysfunction/failure (*n *= 14), central nervous system problems (hemorrhage, ischemia, injury) (*n *= 29), and urgent surgery (*n *= 17). The 'urgent surgery' group included urgent cardiovascular (*n *= 8), visceral (*n *= 6), and orthopedic (*n *= 3) surgery. Due to the small number of patients, the subgroups in 'urgent surgery' were not used for further analysis. The following data were collected from all patients: age, gender, Simplified Acute Physiology Score (SAPS II), length of ICU and hospital stay up to day 28, 28-day survival, and patient location after 28 days. Length of stay (LOS) in hospital before ICU admission (LOS_before ICU_), in hospital (LOS_hospital_), and in ICU (LOS_ICU_) were defined from the patients' records. These data were acquired from the institution's own patient database. LOS_before ICU _was defined as the time from the patient's arrival to the hospital until ICU admission. LOS_hospital _was defined as the time from hospital admission to hospital discharge or 28 days, whichever was shorter. LOS_ICU _was defined as the time in the ICU during the study period. The time a patient stayed in the ICU after a readmission was not added to LOS_ICU_. Three categories were applied for the patient's location: dead, still in hospital after 28 days, or at home/nursing facility. Eight patients were lost to follow-up; consequently, their data could not be used for the assessment of the relationship between ScvO_2 _and outcome.

### Statistical analysis

All data were tested for normal distribution with the Kolmogorov-Smirnov test before further statistical analysis. If the data were normally distributed, parametric tests were used; otherwise, logarithmic or inverse transformation was performed. If the transformation did not result in normal distribution, non-parametric tests were applied. Differences between admission and six hours of ICU stay were assessed using Student's paired *t *test (normally distributed data) and the Wilcoxon signed rank test (otherwise). Differences between the five predefined patient groups (sepsis, *n *= 26; cardiocirculatory dysfunction/failure, *n *= 12; respiratory dysfunction/failure, *n *= 14; central nervous system problems, *n *= 29; and urgent surgery, *n *= 17) were tested with one-way analysis of variance (parametric data) or the Kruskal-Wallis test (otherwise). Receiver operator characteristic (ROC) curves were constructed to identify optimal cutoff values for the association of admission ScvO_2 _and SAPS II, respectively, with 28-day mortality. The optimal cutoff was defined as the value associated with the highest sum of sensitivity and specificity. In addition, univariate analysis was performed to test how 6-hour ScvO_2_, SAPS II, and admission category were associated with 28-day mortality. Differences between patients with high and low admission ScvO_2 _were tested using the *t *test for normally distributed data; otherwise, the Mann-Whitney rank sum test was used. Data on mortality and patient location at 28 days were tested with Fisher's exact test. Data are presented as mean ± standard deviation if normally distributed; otherwise, data are presented as median and range. Statistical significance was assumed at a *p *value of less than 0.05. Sigma Stat version 3.1 (RockWare, Inc., Golden, CO, USA) was used for statistical analysis.

## Results

### Demographic data

Of 349 screened patients, 99 were included in the study. One initially included patient had to be excluded because of a missing ScvO_2 _value at baseline. Table 1 shows the demographic data of all screened patients. Patients included in the study had a higher SAPS II than patients who were screened but not included (43 [11 to 92] versus 29 [6 to 84], *p *< 0.001). For 276 (79%) of the patients who were not included, the reason for exclusion was absence of a central venous catheter within the first two hours after ICU admission, in 38 patients (11%) informed consent was not available, and in 35 patients (10%) other reasons were present. Median (range) age of all included patients was 63 (19 to 83) years, and median SAPS II was 43 (11 to 92) (Table 1). Twenty-nine (30%) of the included patients had diseases of the central nervous system, 26 (27%) had sepsis, 14 (14%) had respiratory failure, 12 (12%) had circulatory failure, and 17 (17%) had undergone urgent surgery. Septic patients had the highest number of organ failures (Table 1). The 28-day mortality was 18% and there were no significant differences between the patient groups. SAPS II was higher in non-survivors than in survivors (70 [47 to 92] versus 39 [11 to 87], *p *< 0.001). Median (range) LOS_ICU _was 3 (1 to 28) days. There was a significant difference in LOS_ICU _in the different patient groups (*p *= 0.038). LOS_hospital _up to day 28 was 19 (1 to 28) days, without differences between the patient groups (Table 1). No difference was seen in LOS_before ICU _in the different patient groups or in patients with an admission ScvO_2 _of less than or equal to 60% or more than 60%. FiO_2 _was not different in patients with an ScvO_2 _of less than or equal to 60% or more than 60% at admission (0.6 [0.3 to 1.0] versus 0.5 [0.3 to 1.0], *p *= 0.378) and did not differ in the different patient groups (Table 1). Univariate analysis revealed a significant association with 28-day mortality for SAPS II (*p *< 0.001).

**Table 1:** Demographic, ScvO_2_, and outcome data

	All patients (*n *= 98)	Cardiocirculatory failure (*n *= 12)	Sepsis (*n *= 26)	CNS disease (*n *= 29)	Respiratory failure (*n *= 14)	Other (*n *= 17)
Median age in years^a^	63 (19–83)	69 (39–79)	65 (35–83)	51 (19–79)	73 (32–83)	70 (28–83)
SAPS II	43 (11–92)	43 (32–89)	45 (11–87)	50 (11–92)	35 (19–86)	34 (13–58)
ScvO_2 _at ICU admission (%)^a^	70 ± 12	60 ± 13	68 ± 12	77 ± 12	64 ± 11	73 ± 9
ScvO_2 _after six hours in ICU (%)^a^	71 ± 10	67 ± 9	67 ± 10	79 ± 7	68 ± 10	68 ± 6
LOS_ICU _in days^a^	3 (1–28)	3 (1–9)	4 (1–25)	3 (1–28)	6 (1–28)	1 (1–10)
LOS_hospital _in days	19 (1–28)	13 (1–28)	28 (1–28)	12 (1–28)	22 (7–28)	18 (5–28)
LOS_before ICU _in days	0.3 (0–38)	0.1 (0–20)	0.8 (0–39)	0.1 (0–20)	2.4 (0–26)	0.5 (0–15)
28-day mortality (%)	18	33	27	24	7	0
FiO_2 _at ICU admission	0.6 (0.3–1.0)	0.7 (0.4–1.0)	0.5 (0.3–1.0)	0.5 (0.3–1.0)	0.8 (0.4–1.0)	0.5 (0.4–0.7)
Number of organ failures	2 (0–4)	2 (1–4)	3 (0–4)^a^	1 (0–3)	2 (0–4)	2 (0–4)
Percentage of patients per group with low (< 60%) ScvO_2 _on ICU admission	21	17	35	3	21	6

^a^Kruskal-Wallis analysis for variance on ranks, *p *< 0.05. Values are expressed as mean ± standard deviation or as median (range). CNS, central nervous system; FiO_2_, inspired fractional oxygen concentration; ICU, intensive care unit; LOS_before ICU_, length of stay in hospital before intensive care unit admission; LOS_hospital_, length of stay in hospital; LOS_ICU_, length of stay in intensive care unit; SAPS II, Simplified Acute Physiology Score; ScvO_2_, central venous oxygen saturation.

### ScvO_2 _in the whole collective and in different patient groups

ScvO_2 _of the whole patient group was 70% ± 12% at ICU admission and 71% ± 10% six hours later (*p *= 0.484; Table 1). There was no overall change in ScvO_2 _between baseline and six hours in either the surviving or non-surviving group of patients. However, there was a significant increase in ScvO_2 _at six hours in the overall group of patients with an ScvO_2 _value of less than 60% at baseline (52% ± 5% to 63% ± 9%, *p *< 0.001). Significantly different ScvO_2 _values at ICU admission were observed in the different patient groups (*p *< 0.001), with the CNS disease group showing the highest mean values (77% ± 12%) and patients with cardiocirculatory failure the lowest mean values (60% ± 13%). Mortality appeared to be highest in the patient group with cardiocirculatory failure, followed by patients with sepsis and patients with diseases of the central nervous system, but without any significant differences between the patient groups (Table 1).

### Systemic hemodynamics in the whole collective and in different patient groups

Table 2 shows the systemic hemodynamic data of the whole collective and of the different patient groups. Mean arterial pressure decreased significantly after six hours of ICU stay in the whole collective. All other recorded parameters in the whole collective and in the patient groups remained largely unchanged.

**Table 2:** Hemodynamic data from the whole collective and the different patient groups

		Systolic blood pressure (mm Hg)		Mean arterial blood pressure (mm Hg)		Heart rate (beats per minute)		Peripheral oxygen saturation (%)		Axillary temperature (°C)	
	*n*	0 hours	6 hours	0 hours	6 hours	0 hours	6 hours	0 hours	6 hours	0 hours	6 hours

All patients	98	120 ± 30	113 ± 23	81 ± 19	76 ± 14^a^	92 ± 24	89 ± 20	97 (56–100)	96 (87–100)	36.8 (33.4–39.1)	37.2 (34.0–39.5)
Cardiocirculatory failure	12	95 ± 23	100 ± 18	70 ± 16^b^	68 ± 9	97 ± 24	87 ± 15	94 (76–100)	98 (91–100)^c^	35.9 (35.3–37.3)	37.0 (36.4–39.0)
Sepsis	26	114 ± 30	106 ± 20	77 ± 20	71 ± 11	99 ± 27	94 ± 25	96 (56–100)	97 (87–100)	36.9 (35.3–39.1)	37.3 (36.0–39.5)
CNS disease	29	139 ± 27^b^	124 ± 27^a,b^	90 ± 18	83 ± 17^b^	84 ± 20	88 ± 18	100 (87–100)	98 (90–100)	36.7 (33.4–39.1)	37.4 (35.6–39.4)^c^
Respiratory failure	14	113 ± 29	116 ± 23	76 ± 17	78 ± 15	99 ± 26	92 ± 25	93 (87–100)^b^	94 (88–99)^b^	36.7 (35.5–37.8)	37.5 (35.9–38.6)^c^
Other	17	119 ± 23	111 ± 18	81 ± 17	75 ± 8	85 ± 17	84 ± 12	99 (88–100)	96 (90–100)	36.2 (34.2–38.1)	37.2 (34.0–38.2)

^a^Paired *t *test, *p *< 0.05; ^b^Kruskal-Wallis analysis for variance on ranks, *p *< 0.05; ^c^Wilcoxon signed rank test, *p *< 0.05. Values are expressed as mean ± standard deviation or as median (range). CNS, central nervous system.

### ROC curve analysis

**Figure 1:**
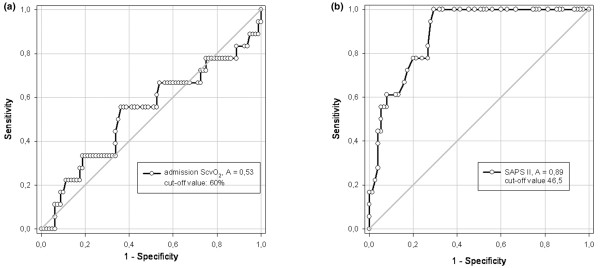
Receiver operator characteristic (ROC) analysis of central venous oxygen saturation (ScvO_2_) and Simplified Acute Physiology Score (SAPS II). Outcome parameter for ROC curves of **(a) **ScvO_2 _and **(b) **SAPS II was 28-day mortality. Area under the curve (A) values were 0.53 for ScvO_2 _and 0.89 for SAPS II.

The whole patient collective was divided into two groups by calculating the optimal cutoff value for admission ScvO_2 _with respect to 28-day mortality using ROC curve analysis. ROC curve analysis revealed two nearly identical optimal cutoff values for association of ScvO_2 _with 28-day mortality (Figure 1a) (ScvO_2 _of 60%: sum of sensitivity and specificity 1.13, and ScvO_2 _of 69%: sum of sensitivity and specificity 1.15). In patients with an ScvO_2 _of less than or equal to 60% at ICU admission, 28-day mortality was higher than in patients with an ICU admission ScvO_2 _of more than 60% (29% versus 17%, *p *< 0.05). In contrast, an ScvO_2 _cutoff value of 69% did not reveal a significant difference in mortality between the groups (21% versus 17%, *p *= 0.701). Accordingly, a cutoff value of 60% was used for further analysis. Those patients in whom the ScvO_2 _value was less than or equal to 60% were defined as patients with a 'low' ScvO_2_, and those with an ScvO_2 _of more than 60% were defined as patients with a 'high' ScvO_2_. For other parameters, ROC curve analysis revealed a difference in mortality only in SAPS II (Figure 1b). The highest sum of specificity and sensitivity was 1.17 at a SAPS II of 46, with a mortality of 38% versus 0%, *p *< 0.001.

### Low/high ScvO_2 _and mortality and LOS

**Figure 2:**
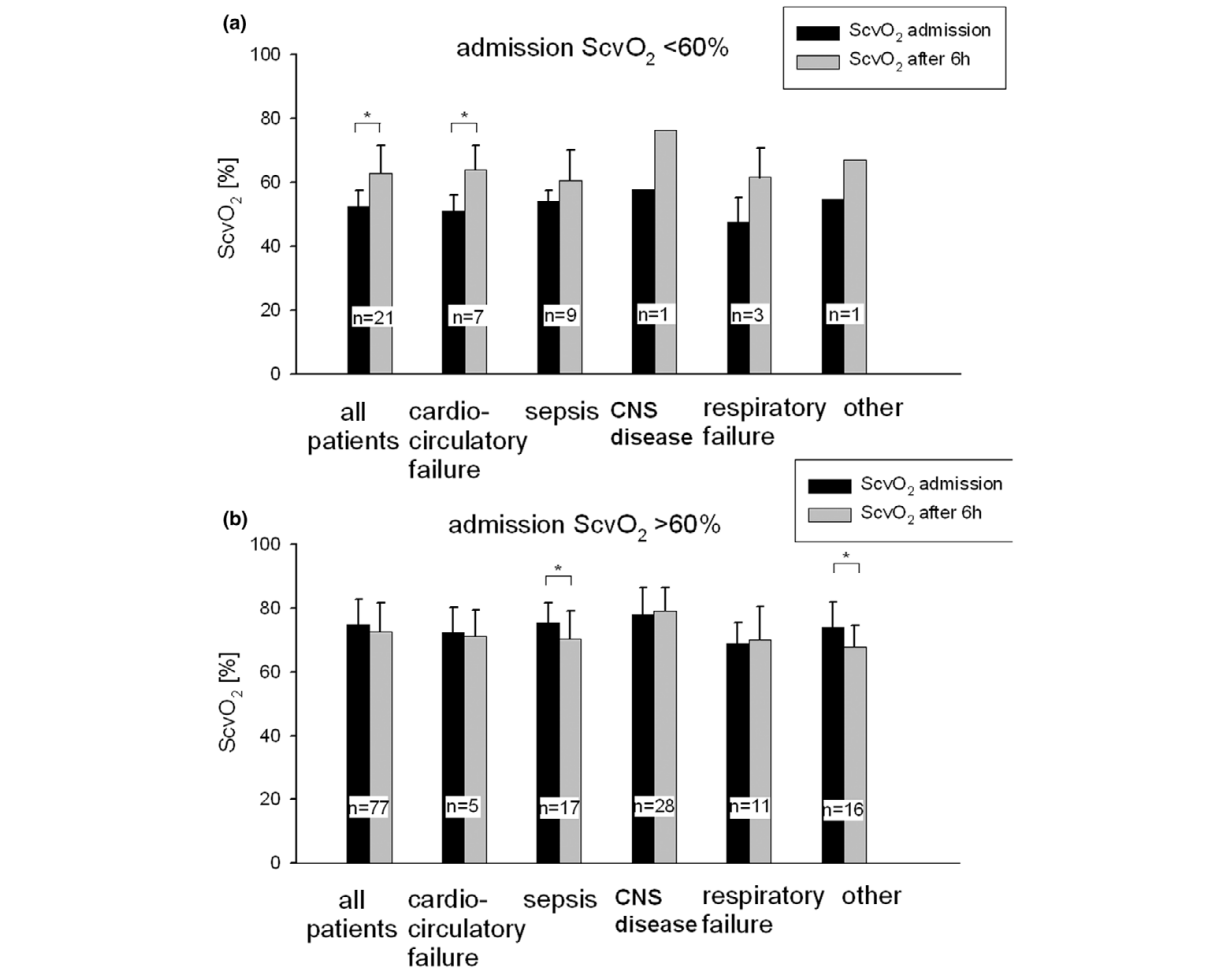
Central venous oxygen saturation (ScvO_2_) trends in the different patient groups. Trends are divided into an intensive care unit admission ScvO_2 _of less than **(a) **and greater than **(b) **60%. ScvO_2 _is displayed as mean ± standard deviation. *Student's paired *t *test, *p *< 0.05, versus admission ScvO_2_. CNS, central nervous system; n, absolute number of patients per subgroup.

Figure 2a,b shows ScvO_2 _at admission and after six hours for the whole collective and for the patient groups with respect to low and high ScvO_2 _values at ICU admission. In patients with a low admission ScvO_2_, ScvO_2 _increased after six hours of ICU stay (52% ± 5% versus 63% ± 9%, *p *< 0.001), whereas no increase was seen in patients with a high admission ScvO_2 _(Figure 2a,b).

LOS_hospital _up to 28 days was not different in patients with low versus high admission ScvO_2 _(18 ± 11 days versus 19 ± 16 days, *p *= 0.971) and did not differ between patient groups (Table 1). Exclusion of non-survivors from LOS analysis did not result in different LOS_hospital _between patients with low (28 [8 to 28] days) and high (21 [1–28] days, *p *= 0.120) ScvO_2_. Similarly, LOS_ICU _was not different between the two groups (4 [1 to 19] days versus 3 [1 to 28] days, *p *= 0.767).

### Outcome

**Figure 3:**
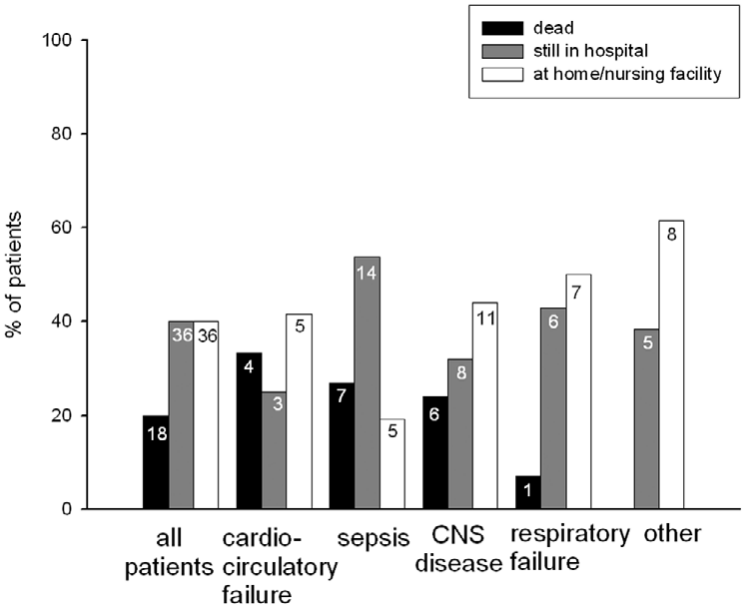
Patient outcome in all patients and in the different patient groups. The numbers in the bars indicate the absolute number of patients per subgroup. Eight patients were lost to follow-up and had to be excluded from outcome analysis. No statistical difference was found for patient location at 28 days in the whole collective and in the different patient groups. CNS, central nervous system.

Figure 3 shows patient location after 28 days. Eight patients were lost to follow-up and had to be excluded from outcome analysis. No significant differences were seen either in the whole collective or in the different patient groups.

## Discussion

The main finding of this study was that low ScvO_2 _at admission was associated with increased risk of mortality in an unselected group of unplanned ICU admissions. The use of ScvO_2 _as a hemodynamic goal is increasingly popular but has not yet been fully evaluated. Only one study has attempted to answer this question in a prospective, interventional manner [13]. Observational studies have described changes in ScvO_2 _in different patient groups. In particular, the prognostic significance of a decrease in ScvO_2 _to less than 65% has been demonstrated in trauma [14], severe sepsis [5,15,16], myocardial infarction [15,17], and cardiac failure [18]. The problems with much of the available observational data are that they are derived mostly from small studies with homogenous patient populations and that these studies did not show an association with outcome parameters such as number of complications, LOS, and/or mortality. In contrast, our study represents a heterogeneous population of a multidisciplinary ICU. Furthermore, we investigated ScvO_2_, not SvO_2_. ScvO_2 _can be measured easily in patients with a central venous line in place, whereas SvO_2 _requires a pulmonary artery catheter. A recent study by Varpula and colleagues [7] showed that SvO_2 _cannot be estimated by ScvO_2 _in patients with septic shock, whereas others showed a close correlation between these parameters [6]. It is noteworthy that ScvO_2 _and not SvO_2 _was implemented in the guidelines of the Surviving Sepsis Campaign. Finally, we have focused on the very early trend in ScvO_2 _in unplanned ICU admissions. In contrast, the study by Krafft and colleagues [5] focused on transient drops in SvO_2_.

ROC analysis for admission ScvO_2 _identified two nearly identical cutoff values for outcome prediction – 60% and 69% – but only the 60% value was significant. This significance was mainly the result of the high specificity (> 80%); that is, the number of survivors with an admission ScvO_2 _of more than 60% was higher than would have been expected from the overall mortality in this population. In contrast, the sensitivity was rather low (33%). With the cutoff value of 69%, both sensitivity and specificity moved closer to 50%. Recently, Pearse and colleagues [12] found that a similar ScvO_2 _cutoff value (65%) was predictive of postoperative complications but not of mortality and LOS in patients undergoing major surgery. Such cutoff values are useful in demonstrating an association between low ScvO_2 _and outcome. In contrast, using any value of a single physiologic variable as a therapeutic target is clearly simplistic and not supported by the present study.

Data on the outcome of critically ill patients with low ScvO_2 _are rare [19], and so far, no study has demonstrated that ScvO_2_-guided treatment can reduce mortality in ICU patients, although LOS has been decreased in cardiac surgery patients using SvO_2 _as a parameter for increasing systemic oxygen supply [3]. In our study, ScvO_2 _was not included as a target parameter for hemodynamic management. The rationale for evaluating ScvO_2 _as a goal in the resuscitation of unplanned ICU admissions is the fact that ScvO_2 _represents the 'oxygen supply reserve' of the region from which the blood is drained. If the central venous line is located in the superior vena cava (as in the present study), this region is head, neck, and upper extremities. Although significant differences between SvO_2 _and ScvO_2 _must be assumed [7], changes in these two parameters seem to occur in parallel [6,11].

Previous studies have suggested that cardiac output is associated with outcome in critically ill patients [20,21]. In our patients, cardiac output data – when measured at all – were not collected. If cardiac function was assessed in the very early phase of ICU admission, it was done by echocardiography and rather qualitatively than quantitatively.

Early goal-directed therapy (EGDT) for patients with severe sepsis or septic shock, which includes treatment goals for mean arterial and central venous pressures and ScvO_2_, has been shown to increase survival [13]. Newer data suggest that 'bundles' of procedures and therapies such as EGDT, recombinant human activated protein C, intensive insulin therapy, and hydrocortisone application may also improve outcome in septic patients [22,23]. The study by Rivers and coworkers [13] predominantly included patients with low ScvO_2 _plus elevated lactate concentrations. Under these conditions, low ScvO_2 _may be a reflection of supply dependency; that is, oxygen delivery does not meet consumptive demands. Because we did not measure lactate concentration systematically, direct comparisons between our results and those of Rivers and coworkers [13] cannot be made.

In the subgroup of septic patients in our study, the ScvO_2 _was relatively high at admission. Furthermore, in septic patients with an admission ScvO_2 _higher than 60%, the ScvO_2 _even decreased (*p *= 0.028). This finding is in contrast to data reported by Rivers and colleagues [13], who found substantially lower ScvO_2 _values in patients with severe sepsis and septic shock, although it must be noted that in their study baseline data were recorded on admission to the emergency department. Whether the early increase in ScvO_2 _in patients with an admission ScvO_2 _of less than or equal to 60% was the result of a changing clinical condition, the treatment, or a combination of both cannot be determined ScvO_2 _was not known by the treating physicians and hence was not a target variable in the treatment of the patients. Three principal mechanisms can explain an increasing ScvO_2 _in critically ill patients: an increase in systemic oxygen delivery, a decrease in systemic or regional oxygen consumption, and blood flow redistribution toward the upper body. Because one of the very first attempts in unplanned admission to the ICU is the establishment of sufficient oxygen transport, increasing oxygen delivery is likely to have contributed to increasing ScvO_2_. This could explain, for example, the increase in ScvO_2 _in patients with cardiocirculatory failure. Sedation and analgesia where needed may have been able to decrease oxygen consumption in some of the patients, whereas in others mitochondrial dysfunction may explain (in theory) an increase in ScvO_2 _as well [24]. Blood flow redistribution is a hallmark of severe sepsis and septic shock. Nevertheless, blood flow redistribution to the upper part of the body seems to be rather unlikely under these conditions. Specifically, in septic patients, ScvO_2 _did not increase during the first six hours in the ICU.

In our group of septic patients, only five were admitted directly to the ICU; the others stayed in the hospital between 90 minutes and 38 days (median 4.5 days) before ICU admission. Nevertheless, the LOS_before ICU _did not correlate with the admission ScvO_2_. The ScvO_2 _values at admission in our study were comparable to the SvO_2 _values reported by Gattinoni and coworkers [25] for septic patients.

### Limitations of the study

A substantial number of unplanned admissions could not be included in this study because they did not have a central venous line in place at ICU admission and were not expected to have an immediate need for one. It can reasonably be assumed that these were most likely patients with a rather normal ScvO_2 _who are now missing from the outcome analysis. This may bias our study toward sicker patients. Nevertheless, patients without central venous lines were missing one of the inclusion criteria. The inclusion criterion 'central venous line in place' selected patients for whom blood samples for the measurement of ScvO_2 _could be obtained, but also to a large extent patients who required a central venous line for hemodynamic resuscitation, and these were the patients we intended to focus on.

Patients with diseases of the central nervous system, especially brain injury, usually have a rather high ScvO_2_, partly due to the disease but certainly also as a result of deep sedation and hypothermia. Despite the fact that in this group of patients in our study a disease of the central nervous system was the leading diagnosis, many of them also had concomitant diseases/injuries. Five of the 29 patients in this group had an admission ScvO_2 _of less than or equal to 70%. The aim of this study was to assess early changes in ScvO_2 _in the heterogeneous group of unplanned ICU admissions. This is what makes our study different from other investigations, such as the study by Pearse and colleagues [12], who have already shown that low ScvO_2 _in a more homogenous patient collective is associated with an increased rate of postoperative complications.

## Conclusion

An ScvO_2 _of less than 60% at ICU admission is associated with high mortality, but not with an increased LOS_hospital_. Standard ICU treatment increased but did not normalize ScvO_2 _in these patients, and this change in ScvO_2 _was not related to outcome. Whether ScvO_2_-guided treatment aiming at higher ScvO_2 _levels improves outcome should be tested in randomized controlled trials.

## Key messages

• ScvO_2 _of less than 60% on unplanned admission to the ICU was associated with high mortality, but not with an increased length of stay in the hospital.

• Standard ICU treatment increased but did not normalize ScvO_2 _in patients with unplanned ICU admissions, and this change in ScvO_2 _was not related to outcome.

## Abbreviations

EGDT = early goal-directed therapy; FiO_2 _= inspired fractional oxygen concentration; ICU = intensive care unit; LOS = length of stay; LOS_before ICU _= length of stay in hospital before intensive care unit admission; LOS_hospital _= length of stay in hospital; LOS_ICU _= length of stay in intensive care unit; ROC = receiver operator characteristic; SAPS II = Simplified Acute Physiology Score; ScvO_2 _= central venous oxygen saturation; SvO_2 _= mixed venous oxygen saturation.

## Competing interests

The authors declare that they have no competing interests.

## Authors' contributions

HB analyzed data, calculated statistics, and wrote the first draft of the manuscript. MH, BJ, NW, FP, and DT screened patients, collected and analyzed data, and revised the manuscript. JT assisted in the analysis and interpretation of data, and revised the manuscript. SMJ designed the study protocol, assisted in the analysis (including statistics) and interpretation of data, and revised the manuscript. All authors read and approved the final manuscript.
